# Exploring Consumers’ Preferences and Attitudes to Honey: Generation Approach in Slovakia

**DOI:** 10.3390/foods12101941

**Published:** 2023-05-10

**Authors:** Peter Šedík, Martina Hudecová, Kristína Predanócyová

**Affiliations:** 1 Institute of Marketing, Trade and Social Studies, Faculty of Economics and Management, Slovak University of Agriculture in Nitra, 949 76 Nitra, Slovakia; martina.hudecova21@gmail.com; 2 AgroBioTech Research Centre, Slovak University of Agriculture in Nitra, 949 76 Nitra, Slovakia; kristina.predanocyova@uniag.sk

**Keywords:** consumer behaviour, honey preferences, generation marketing

## Abstract

Honey is popular among consumers for its composition and healing properties. The aim of the paper is to study the differences in honey preferences across various age generations in Slovakia. The study is based on primary data obtained by conducting an online questionnaire survey on a sample of 1850 Slovak consumers of honey in 2022. Multiple correspondence analyses and non-parametric tests were applied to study the differences in preferences across selected age cohorts (Generation Z, Generation Y, Generation X and Silver Generation). The results show that Silver Generation tends to consume honey due to its nutritional values and prefers to consume monofloral honey of a dark colour, while Generation Z does not use honey in cosmetics or consume it due to its nutritional values and are inclined to prefer polyfloral honey. The utilisation of honey in cosmetics was associated mostly with Generation X. Younger consumers (Generation Z and Generation Y) have a very low awareness of creamed honey and honey with additions in comparison to Silver Generation or Generation X. In addition, the results reveal that propolis, royal jelly and bee pollen were the most attractive additions for honey across all age cohorts in Slovakia, while spirulina and chilli were the least attractive additions.

## 1. Introduction

Recently, health has been recognised as the most significant determinant pushing innovation in the global food and beverage industry [1]. Therefore, organisations have new opportunities to manufacture various nutritionally adjusted food concepts, such as sweeteners and light, fortified, and functional products [2]. Functional foods are gaining popularity, and the functional food market is expanding rapidly, and producers are actively responding by releasing new products that satisfy consumer demand [3,4,5]. 

Functional foods provide nutritional elements that support a healthy lifestyle and even treat some disorders. According to Doyon and Labrecque [6], functional foods provide the body with the vitamins, carbs, proteins, lipids, and other essential nutrients needed for proper functioning. Bee products undoubtedly belong to the category of functional foods. The evidence points to the increasing importance of bee products as a functional food [7]. More specifically, honey is acknowledged as one of the first known functional foods. It has been recognised for centuries for its health benefits [8] and as a ready-to-eat energy source [9]. Honey is the subject of investigation due to its medical, antioxidant and antibacterial capabilities [10]. Moreover, recently, it has been demonstrated that honey is gaining relevance among agri-food products due to many studies investigating the key variables affecting consumers and purchasing behaviour, such as health [11], therapeutic properties [12,13], packaging and quality [14], and organic production and place of production [15,16]. 

This study focuses on finding preferences and attitudes towards honey in generation-al cohorts (Silver Generation, Generation X, Generation Y, Generation Z). It is crucial to understand consumers’ preferences and attitudes in order to create specific marketing strategies applied to different generations. According to the available research, it could be concluded that only a few studies dealt with individual generations—Millennials [17] and Generation Z [18,19,20]. No study has been carried out to explore the preferences and attitudes of individual generations in Slovakia. In order to fill up this scientific gap, this study intends to investigate the differences in consumer preferences for honey across various generations in Slovakia, and the following research questions were developed:

RQ1: Are there any differences in the preferences for honey colour and type across the age cohorts?

RQ2: Are there any differences in the consumer awareness of creamed honey and honey with additions across the age cohorts? 

RQ3: Are there any differences in the preferences for selected types of honey with additions across the age cohorts? 

The study is structured into several parts. The introduction outlines the objectives and emphasises the necessity and significance of the research. The literature review focuses on honey’s functional characteristics, the use and varieties of honey and consumer behaviour towards honey. The following section deals with the methodology, the findings of the results and the discussion. The concluding remarks are presented in the final section.

## 2. Literature Review

### 2.1. Current Use and Variety of Honey

The usage of honey dates back to ancient history. Due to its nutritional and medicinal benefits, honey has been utilised for centuries [21]. It has been consumed in various methods but mainly as a sweetener and flavouring ingredient. It is also known to be a healthier sugar alternative [22]. Moreover, regarding taste, honey can be consumed directly or be part of side dishes. Honey can be found in a wide range of colours and flavours, which is affected by the type of nectar that bees collect from diverse floral sources. Its flavour can also range from delectably mild to noticeably bold. Lighter-coloured honey typically has a milder flavour, whereas darker-coloured honey is typically more robust and stronger and consists of more minerals [23]. There are wide varieties of monofloral and polyfloral honey depending on whether plants arise predominantly from single or several species [24], with the origin having a significant influence on the honey’s composition and sensory qualities. 

### 2.2. Functional Characteristics of Honey

Honey can be marked as a superfood connected with several health properties [25]. According to Qamer, Marghitas and Muhammad [26,27,28], honey contains valuable nutrients, such as physiologically significant amino acids and bioactive substances, such as vitamins, phenols, flavonoids, fatty acids and organic acids, which influence its nutritional value and health-promoting properties. Its valuable role has been confirmed for its antioxidant, antimicrobial and anti-inflammatory effects, improving the immune system [29] and may decrease cardiovascular risk [30]. In addition, studies showed evidence supporting honey as a potential antidiabetic [31] and anticancer agent [32]. Many types of wounds, including burns, surgical sites, infected surgical wounds, chronic ulcers, malignant wounds and newborn wounds, have been successfully treated with honey [33]. In addition, consuming honey also represents a promising cure for several illnesses, including coughs, gastric disturbances and upper respiratory tract infections [34]. This is in line with Al-Hatamleh et al. (2020) [35], who declare that consuming honey may reduce the severity of COVID-19 infection, either directly through antiviral activity against the virus or indirectly through improved immunological function. The successful use of honey has been reported for treating various ophthalmological conditions, such as conjunctivitis and corneal lesions [36].

As mentioned, honey’s functional composition has beneficial health effects. We are currently encountering new products in the honey market. According to Šedík et al. [20], the latest trend driving the honey market is honey with additions. Spices, herbs, dried fruits, pollen, propolis and various favourable ingredients can be added to honey to create new, distinctive products. Moreover, nuts, turmeric, ginger and cinnamon can be added for health properties [37]. As reported by Dżugan et al. [38], new types of honey, such as creamed honey with the addition of dried herbs, flooded the market. Recently, the studies by Tomczyk et al. and Miłek et al. [39,40] focused on rape honey enriched with the fruits of the chokeberry and the fruits and leaves of the Morus alba. Based on the results, adding different plants increased health-promoting aspects depending on the pharmacological characteristics. Grabek-Lejko et al. [3] analysed the biological activities of rape honey enriched with blackberry and raspberry fruits and leaves. Higher antiviral, antibacterial and antioxidant potential has been proven in enriched honey. The increase in the honey’s antioxidant activity also has a minor impact on sensory qualities, as proven in a study by Ćetković et al. [41].

### 2.3. Consumer Behaviour and Preferences for Honey

There are few studies investigating consumer behaviour towards honey in the current literature. A study by Zanchini et al. [11] revealed the main drivers regarding honey consumption: colour, origin and organic certification. The consumption of honey for health reasons was mainly influenced by generation and gender. The health aspect of consumption was also confirmed in research by de Oliveira Neto et al. [42], where Brazilian respondents consumed honey because of its healthiness and tastiness. The findings of Kleisiari et al. [43] revealed that the critical reason affecting European honey consumption is the health impact, which is related to the therapeutic properties and high nutritional value of honey. Moreover, some studies focused on the socio-economic factors affecting honey consumption. Higher honey consumption was associated with medium to high-income levels in a study by Pocol and Teselios [44]. Education, occupation and consumer age were shown to be the most important variables influencing honey intake and purchase in Romania by Pocol and Bolboacă [13].

When purchasing honey, various attributes, such as quality, taste, aroma, the product’s label and the brand’s reputation, come into consideration. Moreover, the region of production is also an essential element regarding honey purchasing [45]. The purchase of local honey has been the subject of various studies. Wu et al. [46] found out that consumers in the USA are willing to pay a higher price for local honey compared to imported honey. Likewise, it has been reported that respondents mostly agreed with statements that purchasing local honey supports local businesses and the economy. Most respondents stated that local honey production is environmentally beneficial to the local community. The preference for local honey was also proven in research in Italy, Serbia and Romania [47,48,49]. Sensory blind testing indicated that only 53% of young consumers showed a preference for local honey in Slovakia [50]. In Hungary, Ványi et al. [51] found out that food safety, organic options and animal welfare awareness were significant determinants of honey purchasing decisions. Furthermore, the findings of Cosmina et al. [52] imply that the organic factor was more significant than the geographical characteristics of the manufacturing area in Italy. These findings are consistent with those of Kehagia et al. [53], who reported that Italian respondents demanded organic honey.

In addition, consumer preferences and behaviour may differ across generations due to the fact that each age cohort of consumers has unique needs, values, desires or opinions; therefore, producers and sellers should not treat them in the same way [54]. Based on the aforementioned, this study aims to identify the differences in honey preferences across various age generations in Slovakia. 

## 3. Material and Methods

### 3.1. Data Collection and Research Design

This study is based on primary research conducted by implementing questionnaire survey. The survey was conducted online using questionnaire created in Google form document. The questionnaire was disseminated via emails and social media platforms (Facebook and Instagram). The data were collected between the period of February 2022 till August 2022. After applying inclusive criteria (honey consumer, age ≥ 18 years and residence in Slovakia), the final research sample included 1850 respondents distributed into four generations more or less equally. The sociodemographic profile is shown in Table 1. Age generations were created based on reviewing the existing studies [54,55,56], and respondents were divided into four generations as follows: Generation Z (born between 2004 and 1997), Generation Y (born between 1996 and 1981), Generation X (born between 1980 and 1972) and Silver Generation (born between 1971 and 1952). 

Questionnaire survey was design based on previous comparative study investigating consumption patterns, preferences and purchasing behaviour [57]. Questionnaire included section with socio-demographic questions, section with purpose of use and section with consumer preferences. For measuring consumer preferences, both rating scale questions and multiple choice questions were used. Consumer preferences for honey type, consistency and colour, as well as preferences for honey with additions and creamed honey, were identified by implementing multiple choice questions or dichotomous questions. Consumer awareness for selected types of honey with additions (flavoured honey) was measured by implementing rating scale questions where respondents used 5-point scale (1 represented extremely attractive, and 5 represented extremely unattractive).

### 3.2. Statistical Analysis

All statistical analyses were carried out using statistical software XLSTAT 2022.4.1 (Addinsoft, NY, USA) with the significance level set to 0.05. Multiple correspondence analysis (MCA) was applied to study differences in consumer preferences across age cohorts in case of multiple choice questions (honey consumption for nutritional values, honey usage in cosmetics, honey type, honey colour, awareness of creamed honey and awareness of honey with additions). MCAs were used in several scientific papers to study consumer preferences [58]. The main focus was on the first two factorial dimensions (F1 and F2) acquired from each MCA due to the fact that those factorial dimensions explain a relevant proportion of the data matrix’s inertia. In addition, Friedman test and the Nemenyi post hoc test were used to analyse consumer preferences for selected types of honey with additions (flavoured honey) overall as well as for each age segment. For the purpose of graphical representation of the results (differences in evaluation), Demsar plots were used. Moreover, statistically significant differences in the evaluation of honey flavours between four generations of Slovak consumers were examined using Kruskal–Wallis test. 

## 4. Results

The results show that there do not exist any differences in preferences for honey consistency across the studied generations. The majority of respondents in all age segments tend to prefer a liquid consistency or do not have a specific preference. In general, the liquid consistency of honey may be associated with its freshness and, therefore, represents a key factor in the decision-making process [59]. 

**Figure 1:**
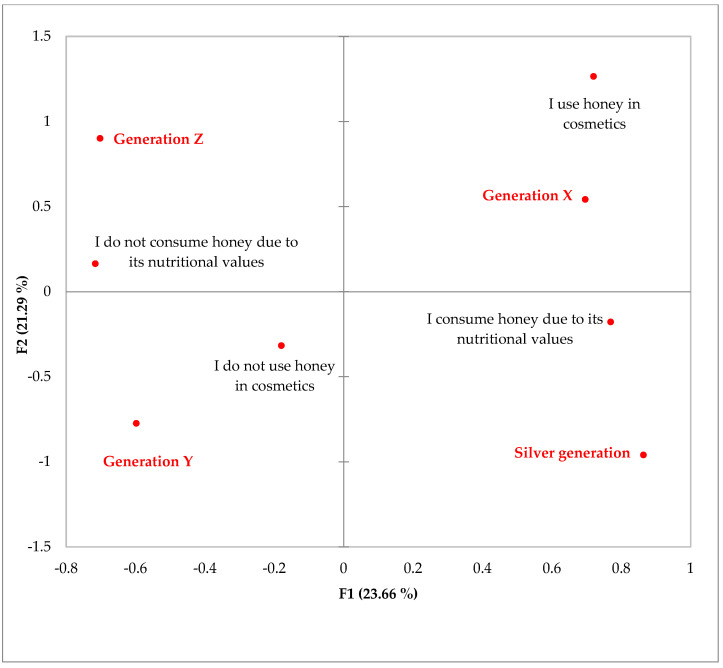
Multiple correspondence analysis (MCA) illustrating two dimensions created using the following questions: age segment, honey consumption for nutritional values as well as honey usage in cosmetics. The last two questions are represented by binary variables (yes/no).

**Figure 2:**
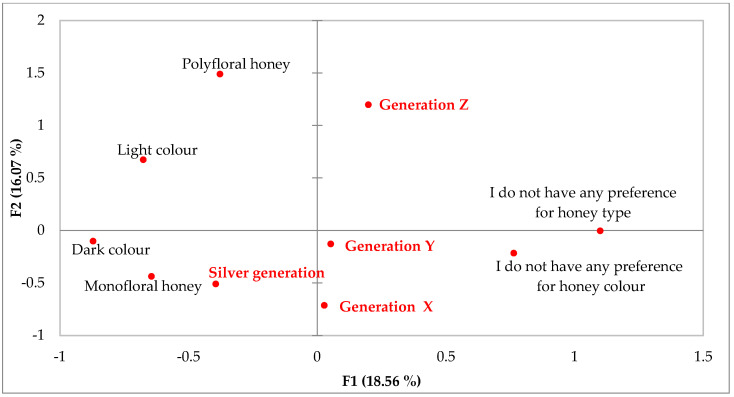
Multiple correspondence analysis (MCA) illustrating two dimensions created using the following questions: age segment and preferences for honey type and colour. Each question is represented by multiple variables.

Moreover, further relationships among selected categorical variables were studied. The results of MCA show that there exists statistically significant differences among generations. Generation X and Silver Generation tend to consume honey more due to its nutritional values as well as use it in cosmetics, while the younger age segments (Generations Z and Y) were associated with negative answers (Figure 1). Other statistically significant differences were acquired among age segments related to honey preferences. Based on MCA, it can be stated that Silver Generation tends to prefer more monofloral honey of a dark colour, while Generation Z prefers polyfloral honey. Generations X and Y were associated with either not having any preferences for honey colour and honey type or were inclined to monofloral honey (Figure 2). 

**Figure 3:**
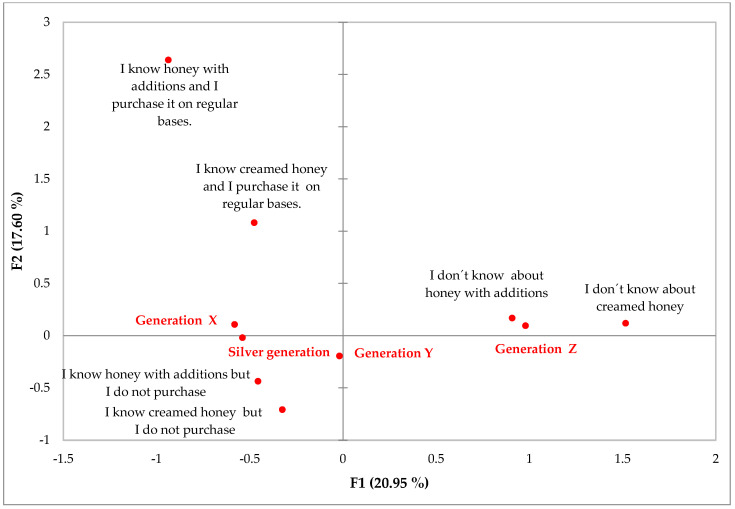
Multiple correspondence analysis (MCA) illustrating two dimensions created using the following questions: age segment, awareness of creamed honey and awareness of honey with additions. Each question is represented by multiple variables.

Furthermore, MCA identified significant differences in the awareness of creamed honey as well as of honey with additions (flavoured honey) across the selected age segments. The results indicate that consumers belonging to Silver Generation tend to know creamed and honey with additions, while Generation Z tends to answer that they do not know both of these honey categories. For Generation X, it can be concluded that this generation tends to know creamed honey, but this segment tends to answer that either they purchase it on a regular basis or do not purchase it. All in all, it can be stated that the younger generations (Generations Y and Z) have lower awareness about creamed honey and honey with additions (Figure 3).

Afterwards, Slovak consumers evaluated 22 selected honey flavours that would be attractive to them to consume. Based on the results of the survey and the mean values of the selected flavours, it is possible to state a positive evaluation of honey with the following flavours: bee pollen (mean = 2.00), propolis (mean = 2.11), royal jelly (mean = 2.18), forest fruit (mean = 2.54), cinnamon (mean = 2.66) and ginger (mean = 2.70). On the other hand, honey enriched with spirulina (mean = 3.74), exotic fruit (mean = 3.52), coconut (mean = 3.52), chilli (mean = 3.61), grapes (mean = 3.51) and cocoa (mean = 3.44) are the least attractive to Slovak consumers.

Furthermore, using the Friedman test, differences in the evaluation of the selected flavours among Slovak consumers were also identified (*p* < 0.0001). Based on Nemenyi’s method, it is possible to point out among which factors exist statistically significant differences (Table 2).

In addition, the consumer attitude and attractiveness of selected flavours in the individual generational segments were evaluated. The results achieved by the consumer survey point to differences in the evaluation of honey flavours between the consumers of individual generations. Statistically significant differences between four generations of Slovak consumers were identified based on the results of the Kruskal–Wallis test in the following flavours: cocoa (*p* = 0.000), ginger (*p* = 0.027), bee pollen (*p* = 0.037), propolis (*p* < 0.0001), royal jelly (*p* < 0.0001), nuts (*p* = 0.017), coconut (*p* = 0.000), strawberries (*p* = 0.003), sea buckthorn (*p* = 0.012), apricot/peach (*p* = 0.024) and exotic fruit (*p* < 0.001).

**Figure 4:**
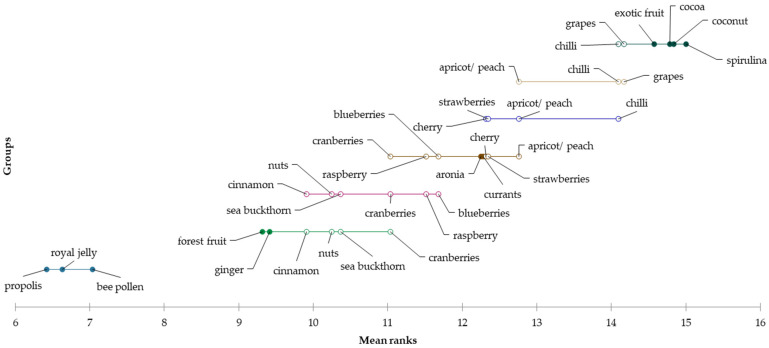
Differences in the evaluation of preferred flavours of honey for the “Silver generation” segment.

According to the mean values, consumers from Silver Generation consider propolis (mean = 1.85), royal jelly (mean = 1.93), bee pollen (mean = 1.99), ginger (mean = 2.68) and forest fruit (mean = 2.66) to be the most attractive honey flavours. In contrast, they would definitely not want to try honey flavoured with spirulina (mean = 3.80), coconut (mean = 3.78), cocoa (mean = 3.75), chilli (mean = 3.64) and grapes (mean = 3.58). Subsequently, using the Friedman test (*p* < 0.0001) and the Nemenyi post hoc test, statistically significant differences in the evaluation of these flavours among consumers of the oldest generation were identified. Demsar plots indicated the results of the Nemenyi test and were used to graphically represent confirmation of the differences in consumer preferences for eating flavoured honey (Figure 4).

**Figure 5:**
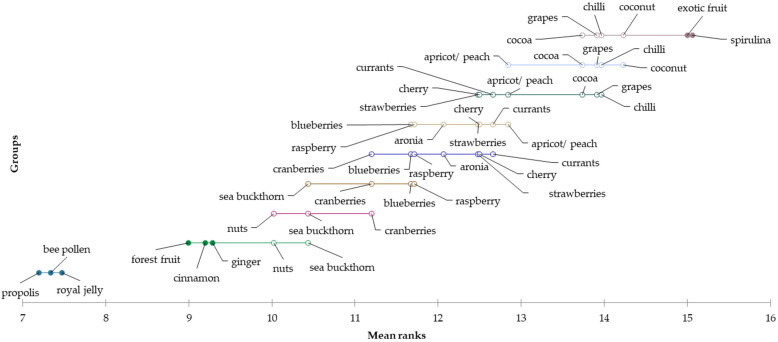
Differences in the evaluation of preferred flavours of honey for the “Generation X” segment.

Furthermore, the attractiveness of the selected flavours was also examined for Generation X. Based on the mean values, it can be concluded that honey with the flavours of propolis (mean = 2.05), bee pollen (mean = 2.07), royal jelly (mean = 2.09), forest fruit (mean = 2.55), ginger (mean = 2.56) and cinnamon (mean = 2.57) are acceptable for Slovak consumers of Generation X. On the other hand, consumers of this generation would not choose honey with the flavours of exotic fruit (mean = 3.75), spirulina (mean = 3.74), coconut (mean = 3.60), chilli (mean = 3.56), nuts (mean = 3.55) and cocoa (mean = 3.52). Statistically significant differences were also identified in the evaluation of flavours among Generation X consumers using the applied Friedman test (*p* < 0.0001). The subsequent Nemenyi post hoc test was used to investigate between which flavours the mentioned differences existed. The Demsar plot (Figure 5) graphically shows the differences in the evaluation of flavours between Slovak consumers of the X generation.

**Figure 6:**
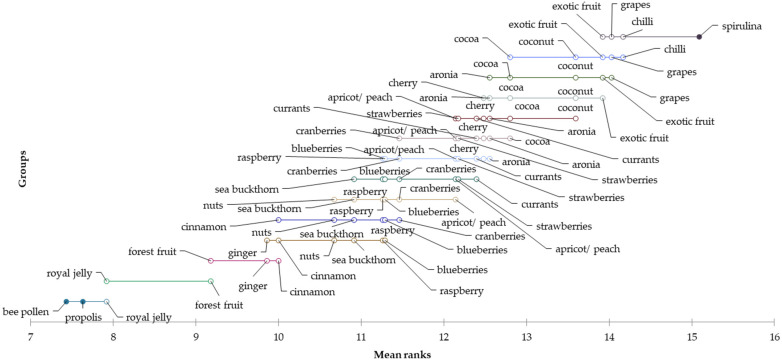
Differences in the evaluation of preferred flavours of honey for the “Generation Y” segment.

According to the mean values, honey with the flavours of bee pollen (mean = 2.11), royal jelly (mean = 2.12), propolis (mean = 2.14), forest fruit (mean = 2.55) and ginger (mean = 2.69) appear to be the favourite among consumers of Generation Y. The least preferred flavours of honey for consumption are spirulina (mean = 3.77), chilli (mean = 3.59), grapes (mean = 3.54), exotic fruit (mean = 3.51), coconut (mean = 3.47) and cocoa (mean = 3.31). Differences in the evaluation of these flavours among consumers of generation Y were explored using the Friedman test (*p* < 0.0001) and the subsequent Nemenyi post hoc test. A graphic representation of the differences is shown using a Demsar plot in the following Figure 6.

**Figure 7:**
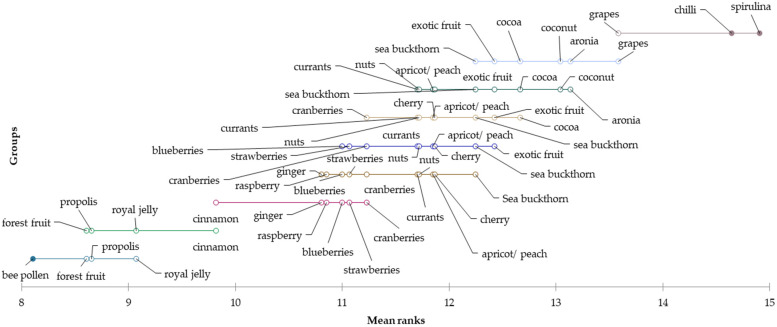
Differences in the evaluation of preferred flavours of honey for the “Generation Z” segment.

The last evaluated segment was Generation Z. In general, it is possible to state a neutral evaluation of the selected flavours among consumers of this generation. However, according to the mean values, it can be concluded that the most positively perceived flavours are bee pollen (mean = 2.20), propolis (mean = 2.32), royal jelly (mean = 2.41), forest fruit (mean = 2.41) and cinnamon (mean = 2.66). The consumers of Generation Z considered the flavours of chilli (mean = 3.56) and spirulina (mean = 3.67) as the least acceptable for consumption. Even in this generation, there were statistically significant differences in preferences for the consumption of flavoured honeys using the Friedman test (*p* < 0.0001) and the subsequent Nemenyi post hoc test. A graphical representation of the differences is shown using a Demsar plot (Figure 7).

## 5. Discussion

The results of the consumer study show statistically significant differences in preferences for the type and colour of honey. The results show that Silver Generation has a strong preference for monoflower types of honey with a dark colour, while Generations X and Y do not have a clear preference and consumers of the youngest Generation Z tend to prefer multiflower honeys. The results can be compared with other studies conducted abroad. The results of the study carried out by Kopała et al. [60] showed that elder respondents consume more often honey compared to younger generation of consumers. Many conducted studies pointed to consumer preference for the type of honey, and based on the results, it can be concluded that polyfloral and multifloral honey [60,61,62], acacia honey [61,62,63,64], lime honey [60,62] and other rare types, such as honeydew and mountain flowers [65,66], are the most preferred among consumers. Moreover, Żak [67] examined honey colour preference and found that young consumers prefer bright honey, and older ones would rather consume honey with a dark colour. Based on the above, it is possible to state identical elements in the preferences for the consumption of honey in terms of colour and type between Slovak consumers and consumers from other countries.

The results of this consumer study conducted in Slovakia show that consumers prefer the liquid consistency of honey, which can be justified by the fact that consumers consider it fresh but also due to easier handling or a low preference for crystallised honey. The above results are confirmed by other studies that showed a high preference for liquid honey consumption [68,69]. Our results also show differences in preferences for the purpose of honey consumption. The older generation of consumers tends to consume more honey mainly because of its nutritional benefits as well as its possible use in cosmetics. This can be justified by the fact that the older generation of consumers has a stronger habit of consuming honey, which has been presented as healthy for many years. Studies aimed at examining the differences in the purpose of honey usage between age generations of consumers are absent; however, Kowalczuk et al. [67] identified that most consumers consume honey for culinary, cosmetic and medicinal purposes. In connection with the above, Kowalczuk et al. [67] divided consumers into three groups based on their use of honey. The first segment of consumers named “Honey eaters” uses honey mainly for culinary purposes. The second group of consumers uses honey rarely in all analysed aspects. These consumers were named “Honey rarely users” and included mainly young consumers. The last group “Honey users” most often uses honey for cooking, cosmetic purposes and the prevention and treatment of various diseases. However, Kleisiari et al. [43] state that the key reason for honey consumption for European consumers is the health impact related to the therapeutic properties and high nutritional value of honey. In this context, it is possible to point to the fact that the level of nutritional knowledge has an impact on consumer behaviour related to honey consumption, and consumers with a better health status consider nutritional and health benefits as a significant motive for honey consumption [62,67]. The results of previous studies confirm our assumptions that honey is presented as a healthy food, and this is the key motive for consumption.

This consumer study in Slovakia also dealt with the consumption and preference of creamed honey and flavoured honey. The results show that Generations Y and Z have low awareness about creamed honey and honey with additions, which could be justified by the fact that younger consumers are indifferent towards honey in general [70]. In the context of the above, Sparacino et al. [71] add that flavour is a very important attribute for the consumption of honey. Mateescu et al. [72] state that a flavoured creamed honey is a good choice for consumers who are interested in consuming a spreadable product with an original flavour and nutritional benefits. Leaka et al. [73] found that in addition to plain honey, consumers also prefer health-enriched and flavoured honeys for consumption. It can be concluded that these additions to honey create additional value compared to the original value. The results of our consumer study show statistically significant differences in the evaluation of honey flavour preferences between all age generations, but in general, it can be concluded that propolis, royal jelly and bee pollen are considered the most preferred honey flavours for consumption by all generations, and the least attractive is spirulina. Consumer attitudes towards individual flavour preferences can be connected to their popularity among consumers. Flavours that consumers have tasted and are familiar with may be more attractive additions compared to those with which consumers have no experience. Moreover, Šedík et al. [54] examined the preference of flavoured honeys among consumers of different age generations. The results show that honey with cinnamon, cacao and coconut are the most preferred by consumers of Generation Z. Generation Y shows a strong preference for honey with nuts, pollen, honeycomb or honey with cinnamon. Of the flavoured honeys, consumers of Generation X prefer honey with pollen and honey with honeycomb. Consumers of the Baby Boomers Generation tend to consume honey with ginger and honey with nuts. Furthermore, Leaka et al. [73] emphasise that consumers prefer honey with the addition of dried fruit or flavoured honeys, such as lemon or cinnamon. However, other honeys are also available on the market, e.g., Eucalyptus honey, Jamun honey and Tulsi honey. Creamed honey flavoured with essential oils can be used to provide a wider and more effective range of health benefits [72]. Based on the above, it can be concluded that flavoured honey is an increasingly popular product among consumers. Enriching honey with other healthy ingredients, such as spices, herbs, dried fruits, pollen, propolis, coumarin and spirulina, can be considered novel innovative food products [20,40,74,75,76].

Based on the comparison of the results of the consumer study conducted by us in Slovakia with the results of other consumer studies conducted abroad, it is possible to conclude that our study brings new results. The novelty is demonstrated in the identification of honey consumption preferences from the point of view of the purpose of use, colour, type, consistency and enrichment of honey with new additives and points to generation differences in the evaluation of these preferences. In addition, the presented study is aimed at evaluating the consumer acceptability and attractiveness of more honey flavours compared to previous studies.

## 6. Conclusions

Understanding consumer preferences is essential for every business, including beekeeping and the honey industry. The research reveals important differences in preferences for honey colour, type and its utilisation across selected age segments (Generation Z, Generation Y, Generation X and Silver Generation). Furthermore, the results show differences in the awareness of creamed honey and honey with various additions, as well as reveal which additions are more attractive or unattractive for each generation. In general, spirulina was evaluated as the least attractive, while other bee products, such as propolis, royal jelly and bee pollen, obtained the highest rating in attractiveness. 

The study’s main managerial implications concern consumer preferences for honey and honey with different additions across various age cohorts in the Slovak honey market. The obtained results provide important data for the honey industry from several points of view. Firstly, knowledge about different preferences for honey and its purpose of use across different age cohorts allows producers (beekeepers and other entities in the honey industry) to implement more effective marketing strategies by altering products and marketing communication to the personal needs and desires of customers. Secondly, this study provides important insights into consumer preferences for honey with additions (flavoured honey) which is nowadays perceived as a product with added biological benefits or a functional product. Creamed honey and honey with additions should be promoted more among younger honey consumers. In addition, the aforementioned results provide important data for new product development in this product segment.

The main limitation of this study is the application of self-reported measures and subjective evaluations and the fact that the survey was conducted only in an online version. Another limitation is connected with the territorial scope of the study, which was applied only at the national level; therefore, future research should be oriented on comparative consumer studies at an international level to identify similarities and differences in consumer preferences for honey and honey-related products.

## Figures and Tables

**Table 1:** Socio-demographic profile of research sample.

Socio-Demographic Characteristics		(%)
Gender	male	34.00%
	female	66.00%
Age cohorts	Generation Z	27.03%
	Generation Y	27.03%
	Generation X	27.03%
	Silver Generation	18.92%
Education	primary	1.30%
	secondary	50.54%
	university	48.16%
Residence	rural area	40.22%
	urban area	59.78%
Economic status	employed	52.8%
	student	17.7%
	entrepreneur	13.9%
	maternity leave	6.6%
	pensioner	5.9%
	unemployed	2.6%
	other	0.4%

**Table 2:** Differences in the evaluation of selected honey flavours.

Sample	Mean of Ranks	Groups								
bee pollen	7.513	A								
propolis	7.563	A								
royal jelly	7.868	A								
forest fruit	9.003		B							
cinnamon	9.718		B	C						
ginger	9.876			C						
nuts	10.700				D					
sea buckthorn	11.041				D					
cranberries	11.248				D	E				
raspberry	11.321				D	E				
blueberries	11.388				D	E				
strawberries	11.992					E	F			
currants	12.258						F			
cherry	12.290						F			
apricot/peach	12.369						F			
aronia	12.523						F			
cocoa	13.394							G		
coconut	13.852							G	H	
grapes	13.904							G	H	
exotic fruit	13.935							G	H	
chilli	14.229								H	
spirulina	15.016									I

Note: flavours with different superscript were evaluated differently from statistical point of view.

## Data Availability

The data presented in this study are available on request from the corresponding author.
